# Laparoscopic ovarian transposition prior to pelvic irradiation in a young female patient with advanced rectal cancer

**DOI:** 10.1186/s40792-015-0119-0

**Published:** 2015-11-12

**Authors:** Kyoichi Kihara, Seiichiro Yamamoto, Taihei Ohshiro, Shin Fujita

**Affiliations:** Colorectal Surgery Division, National Cancer Center Hospital, 5-1-1 Tsukiji, Chuo-ku, Tokyo, 104-0045 Japan; Department of Surgery, Tottori Prefectural Central Hospital, 730 Ezu, Tottori, 680-0901 Japan; Department of Digestive Surgery, Hiratsuka Municipal Hospital, 1-19-1 Nambara, Hiratsuka, 254-0065 Japan; Department of Surgical Oncology, Nagoya University Graduate School of Medicine, 65 Tsurumai-cho, Syouwa-ku, Nagoya, 466-8560 Japan; Department of Colorectal Surgery, Tochigi Cancer Center, 9-13 Yonan 4-Chome, Utsunomiya, 320-0834 Japan

**Keywords:** Ovarian transposition, Rectal cancer, Radiation, Laparoscopy, Fertility, Menopause, Ovarian failure

## Abstract

In the report, we describe the first case of laparoscopic ovarian transposition prior to pelvic radio-chemo therapy in a young female patient with advanced rectal cancer in Japan. A 14-year-old female visited a hospital because of consistent diarrhea and melena. Colonoscopy examination showed a bulky tumor of the rectum, which was diagnosed as moderately to poorly differentiated adenocarcinoma. The diagnosis was cT3N2aM1a (due to lymph node in pelvic side wall), cStage IVA. In an attempt to improve local control and sphincter preservation, neoadjuvant concurrent radio-chemo therapy was planned. Considering that pelvic irradiation particularly in young female might cause ovarian failure, laparoscopic ovarian transposition was carried out prior to pelvic irradiation. Sequentially the patient underwent low anterior resection of the rectum and lymphadenectomy including pelvic side wall. The menstruation was maintained with delay for 6 months after adjuvant chemotherapy. There is no evidence of cancer recurrence at 3 years after the surgery.

In premenopausal patients with rectal cancer undergoing pelvic irradiation, laparoscopic ovarian transposition is one of the choices to prevent ovarian failure.

## Background

As survival rates of colorectal cancer (CRC) improve and more women delay their childbearing years until their 3rd decade, it is evident that preserving fertility in these patients is becoming increasingly important [1–4]. Ovarian transposition (OT), also known as oophoropexy, is a surgical procedure that relocates the ovaries out of the radiation field. First described in 1958, it was initially performed at laparotomy in a uterine cervical cancer patient to preserve ovarian function [5]. Here, we report a case of rectal cancer in a young female patient, in whom laparoscopic ovarian transposition (L-OT) prior to neoadjuvant radio-chemo therapy was carried out to prevent ovarian failure.

## Case presentation

**Fig. 1:**
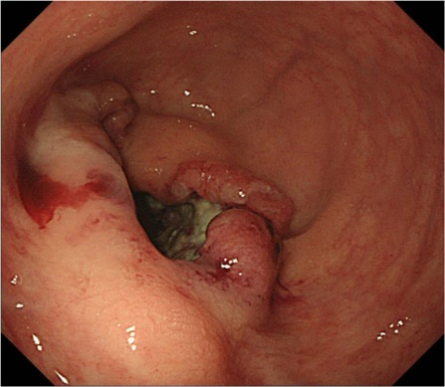
The colonoscopy revealed a Type 2 lesion. The anal margin of the tumor was below the middle transverse rectal fold

**Fig. 2:**
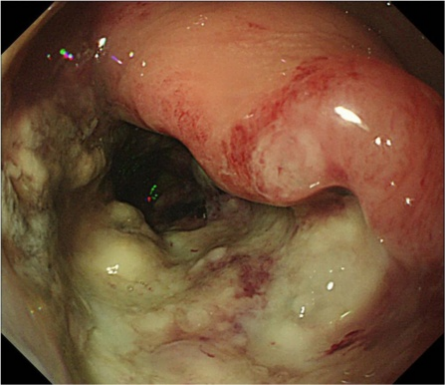
The colonoscopy showed a long cancer canal

**Fig. 3:**
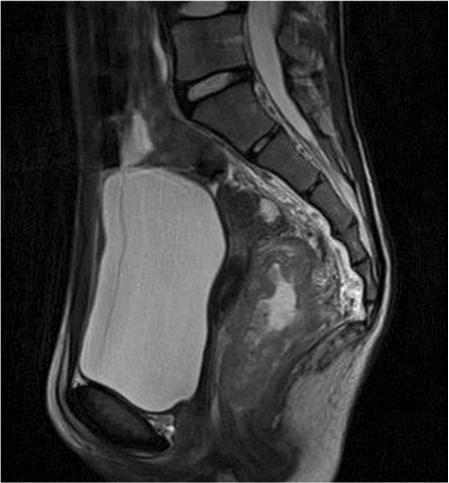
MRI of sagittal plane revealed a balky tumor in lower rectum, measured 76 mm in diameter

**Fig. 4:**
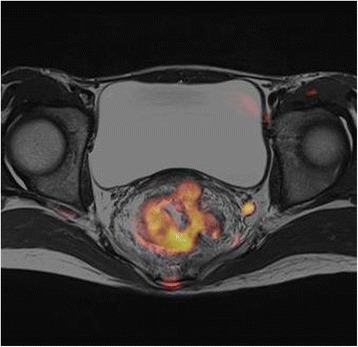
The lymph node in the left pelvic wall was swelling and positive for contrast enhancement

A 14-year-old female visited a local hospital because of persistent diarrhea and blood in her stool. Colonoscopy showed a bulky tumor in the lower rectum (Figs. 1 and 2). The distance between the anal verge and the lower edge of the tumor was 3 cm. The biopsy specimen revealed moderately to poorly differentiated adenocarcinoma. The patient was referred to the National Cancer Center Hospital. There was no remarkable medical history in her past, no malignancy in her close relatives. Carbohydrate antigen 19–9 level rose up to 551 U/ml (<37), while the carcinoembryonic antigen was 2.4 ng/ml (<5.0). The rest laboratory examinations were within the normal range. Magnetic resonance imaging revealed the tumor of the rectum, 76 mm in diameter (Fig. 3), and detected several swelling lymph nodes in mesorectum and left pelvic side wall, which were rational for diagnosing as metastasis (Fig. 4). The clinical diagnosis was cT3N2aM1a (due to lymph node metastasis in pelvic side wall), cStage IVA according to the TNM classification (the 7th edition). In an attempt to improve local control and sphincter preservation, preoperative concurrent radio-chemo therapy was planned. Considering that pelvic irradiation particularly in young female might cause ovarian failure, the patient underwent L-OT before the pelvic irradiation to prevent direct radiation injury to the ovaries.

**Fig. 5:**
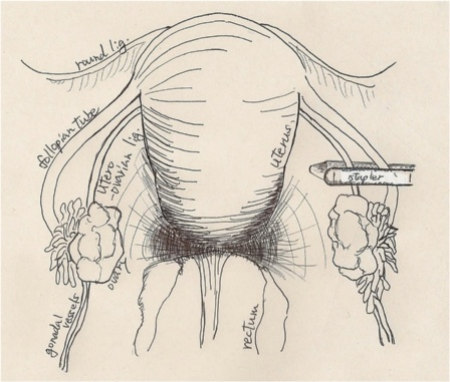
Utero-ovarian ligament was transected the adnexa of uterus by stapling device

**Fig. 6:**
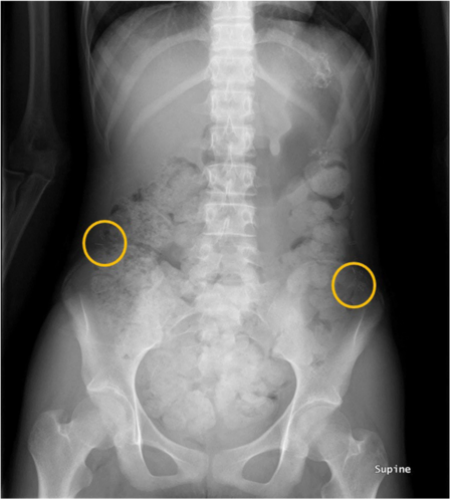
Postoperative roentgenogram showing the position of metallic clips applied to each ovary

**Fig. 7:**
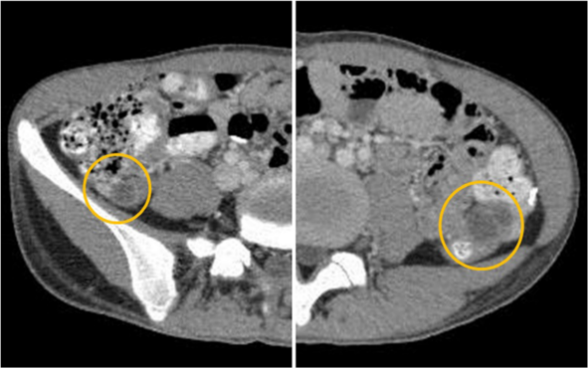
CT showed the ovaries transposed to the level of the anterior superior iliac spine, anterior to the psoas muscle

The procedures were carried out under general anesthesia. A 12-mm port was inserted supra-umbilicus using open technique. After insufflation of abdominal cavity, two 5 mm ports were inserted under direct vision into both sides of hypogastric area. The patient was placed in Trendelenburg’s position to free the pelvis and enable visualization of the genital tract. Small amount of ascites was collected for cytology, and it revealed negative for cancer cell. The right mesocolon and the mesosigmoid to the mesorectum in the left were separated from the underlying retroperitoneum to expose the ovarian vessels and ureters. Stapler was used to divide the utero-ovarian ligament, and the ovarian vessels were mobilized carefully (Fig. 5). The ovary was transposed to the level of the anterior superior iliac spine, anterior to the psoas muscle, and anchored to the peritoneum. Clips were placed to ensure visualization on plain abdominal radiographs (Figs. 6 and 7). The duration was 1 h and 25 min, and the loss of blood was less than 10 ml.

**Fig. 8:**
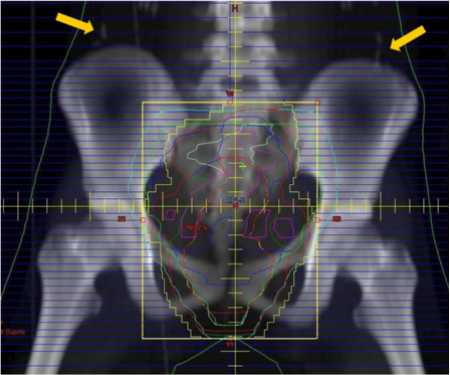
The clips attached to the ovaries (*yellow arrow*) were relocated outside of the radiation field

**Fig. 9:**
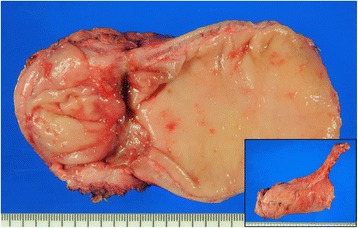
The resected specimen of total mesorectal excision. The tumor shrank and turned to be a depressed scar

A total of 50.4 Gy given in 28 fractions was delivered to the pelvis (Fig. 8), with concurrent biweekly fluorouracil and oxaliplatin. The patient had a partial response to neoadjuvant radio-chemo therapy, and carbohydrate antigen 19–9 level decreased to 34 U/ml (<37). The distance between the anal verge and the lower edge of the tumor decreased to 2 cm after neoadjuvant radio-chemo therapy. Total mesorectal excision, lymphadenectomy of pelvic side wall, and diverting ileostomy were performed after 7 weeks from the completion of neoadjuvant therapy (Fig. 9). The postoperative course was uneventful. Histological examination of the resected specimen revealed moderately to poorly differentiated adenocarcinoma including mucinous component. Infiltration of vessels and nerves was also observed. Final diagnosis was pT3N1bM0, pStage IIIB. The tumor regression was grade 2, according to the definition by the American Joint Committee on Cancer. The patient received eight courses of FOLFOX as adjuvant chemotherapy and underwent stoma closure. She experienced a return of menses with 6 months duration after adjuvant chemotherapy. She has now been followed up for 3 years after surgery with no evidence of tumor recurrence.

### Discussion

We have reported the case of young female, who maintained menstrual cycles following a multidisciplinary therapy for advanced rectal cancer including L-OT prior to concurrent pelvic irradiation and chemotherapy. Recent analyses have indicated that the incidence of CRC in women younger than the age of 40 years was 3–6 % and keep increasing in the western countries [6]. In Japan, CRC is now the second most commonly diagnosed cancer and the most leading cause of cancer-related deaths for women according to the cancer statistics released by Center for Cancer Control and Information Services, Japan [7, 8].

Our case was sporadic early-onset without an apparent predisposing condition for CRC. Sporadic early-onset CRC frequently displays adverse histologic features, such as signet ring cell and mucinous differentiations, and perineural and venous invasion. Probably because of these features, they tend to have a poorer outcome than adult population [9, 10]. As a result of its rarity, there has been no prospective study, targeting young CRC patients, including children. Therefore, the therapeutic strategy for young CRC patients is primarily extrapolated from adult trials. Neoadjuvant radio-chemo therapy is getting to be used in lower rectal cancer to reduce local recurrence and attempt sphincter preservation [11, 12]. As survival rates of CRC improve and more women delay their childbearing years until their 3rd decade, it is evident that preserving fertility in these patients is becoming increasingly important [1–4]. A retrospective study in women, aged 40 years and younger with CRC, has reported that the incidence of amenorrhea in colon and rectal cancer patients were 4.2 % (3 of 72) and 94.1 % (48 of 51), respectively [13]. This data strongly supports that irradiation to the pelvis causes severe damage to the ovaries, and young female patients, who are scheduled to undergo pelvic irradiation, should be counseled regarding fertility preservation options. However, physicians tend to focus on major surgical complications, tumor recurrence, and standard complications of radio-chemo therapy. They are not likely to address fertility concerns with their patients before cancer treatment. Unfortunately, only 15 % of women aged 18–45 years with CRC received pretreatment fertility counseling and nearly 40 % of the women had documented difficulty with pregnancy or changes in menses after treatment [14].

In our multidisciplinary team conference including pediatric oncologists and radiologists, ovarian failure due to pelvic irradiation was concerned. The patient of our case was 14 years old and still in the age of puberty. Gonadal hormones from ovaries were supposed to have important roles not only in fertility but also in physical growth. Many procedures that previously required a laparotomy are now done by laparoscopy. L-OT has been established as a simple and reliable method with reduced morbidity with Hodgkin’s disease and cervical cancer patients [15]. However, there are no studies in CRC patients that address the effects of OT, on female fertility at present. In spite of many cases of OT in the other cancer patients, very limited cases of OT in rectal cancer patients have been reported by a literature search of PubMed by cross-referencing the terms “ovarian transposition” and “rectal cancer.” There are only 31 cases, including our case, that underwent OT in CRC (Table 1) [16–24]. All the cases had chosen laparoscopic approach. The patients’ ages ranged from 14 to 38. Most of them had been diagnosed as T3 and N1 in TNM classification. The case of T1bN0M0, reported by Tulandi [16], was an exceptional case that “transanal resection” had been performed for cT1 lesion initially, and it revealed pT1b. Instead of an additional lymphadenectomy, irradiation was held.

**Table 1:** Summary of reported cases of ovarian transposition for rectal cancer

Author-year	Age	TNM (stage)	Total dose	Chemotherapy	Menstruation (follow-up period)	Pregnancy
Tulandi 1998 [16]	34	T1bN0M0	45Gy/25fr	No	+	+^a^
Bisharah 2003 [17]	28	NA	45Gy/25fr	No	+	
Farber 2005 [18]	28	T4aN0M0	45Gy/25fr	5-FU + LV	+	
Kurt 2007 [19]	24	T3N1M0	45Gy/25fr	5-FU + LV	+^b^	+^c^
Elizur 2009 [20]	28	T3N1M0	NA	No	NA	
	29	T3N1M0		5-FU		+^d^
	33	T1NxMx		5-FU		
	34	T3cN1M0		5-FU		
	38	T3N0M0		5-FU		
Al-Badawi 2010 [21]	23	T3N1M0	NA	No	Lost (10 months)	
	23	TXN1M0		No	+ (60 months)	
	26	T3N1M0		No	+ (46 months)	
	28	T3N1M0		No	Lost (12 months)	
Gareer 2011 [22]	15				+	
	17				+	
(Needle oophoropexy)	19				+	
	20				+	3 of 10 achieved pregnancy
	25	NA	NA	NA	+	
	26				+	
	26				Lost	
	30				+	
	31				+	
	33				+	
Al-Asari 2012 [23]	21				Two of three maintained their menstruation^e^	
	21-27	NA	NA	NA		
	27					
Barahmeh 2013 [24]	33	cT3N1M0	50.4Gy/28fr	5-FU	Lost	
	NA	NA			+	
	NA	NA			+	
	NA	NA			+	
Present case	14	pT3N1bM0	50.4Gy/28fr	5-FU + OX	+ (24 months)	

^a^Two years after surgery [33]

^b^Eight weeks after the completion of the CRT

^c^In utero exitus

^d^Delivered a child 2 years after her operation

^e^One of them resumed menstruation with 4 months delay, resumed menstruation with 6 months delay

Several factors have been identified as significant determinants of ovarian failure. Age at the time of irradiation is known to be an important factor determining the success of OT, so that OT is generally recommended in patients who are less than 40 years of age [25]. Lower and increasingly fractionated doses raise the possibility of repair of the damaged follicular population [26]. Permanent ovarian failure occurs on exposure of 20 Gy of pelvic irradiation in women less than 40 years [27]. The gonadal vascular damage among the procedures and the distance of the transposed ovaries from the radiation field are also very important [28, 29].

OT is not always enough to achieve fertility preservation. Five patients out of 31, in previous rectal cancer cases underwent L-OT, had experienced menopause after pelvic irradiation. It also threatens the other genitals in pelvis. The human oocyte is very sensitive to radiation, with an estimated median lethal dose of less than 2 Gy [30]. The estimated dose of radiation at which half of the follicles are lost in humans is 4 Gy [31]. Exposure to the uterus must be considered in fertility as well; exposure of the prepubertal uterus to doses between 14 and 30 Gy are likely to result in poor uterine growth and uterine dysfunction [32]. There are few literatures about endometrial activity after pelvic irradiation, but as a general rule, a dose of 40 Gy is considered sufficient to destroy the normal endometrium [33]. We have laid much emphasis on preservation of ovarian function, and radiation field including the uterus was planned to improve curability for cancer in this case. Preserving fallopian tubes is technically complex so that they have been resected through the procedure dividing adnexa of the uterus. When the patient desires to have a child, in-vitro fertilization must be considered. Instead of these damages in genitals, it is surprising that six patients out of 31 cases had been pregnant.

Chemotherapy constitutes a significant cytotoxic risk to the ovaries; it can also result in premature ovarian failure. Additionally, 5-FU has almost no effect on human reproductive function, but oxaliplatin induces cellular apoptosis. It therefore has moderate gonadotoxic effects and can cause ovarian failure and birth defects [34]. The clinical diagnosis of the patient was cT3N2aM1a (due to lymph node metastasis in pelvic side wall), cStage IVA. Oxaliplatin was added to the treatment regimen because it may improve disease-free survival in patients with advanced CRC [35–37]. However, it goes without saying that radiation plus oxaliplatin may cause severe or complete ovarian failure. We believed that the risk and degree of ovarian failure might be decreased by performing OT.

## Conclusions

In conclusion, L-OT is one of the choices to prevent ovarian failure and infertility in premenopausal patients of rectal cancer, who are scheduled to undergo pelvic irradiation. Further well-designed studies regarding this issue are warranted.

## Consent

Written informed consent was obtained from the patient for publication of this case report and any accompanying images.

## Abbreviations

CRC: colorectal cancer
OT: ovarian transposition
L-OT: laparoscopic ovarian transposition
